# Sleep Restriction Increases the Risk of Developing Cardiovascular Diseases by Augmenting Proinflammatory Responses through IL-17 and CRP

**DOI:** 10.1371/journal.pone.0004589

**Published:** 2009-02-25

**Authors:** Wessel M. A. van Leeuwen, Maili Lehto, Piia Karisola, Harri Lindholm, Ritva Luukkonen, Mikael Sallinen, Mikko Härmä, Tarja Porkka-Heiskanen, Harri Alenius

**Affiliations:** 1 Brain and Work Research Centre, Finnish Institute of Occupational Health, Helsinki, Finland; 2 Unit of Excellence for Immunotoxicology, Finnish Institute of Occupational Health, Helsinki, Finland; 3 Department of Physiology, Institute of Biomedicine, University of Helsinki, Helsinki, Finland; 4 Centre of Excellence for Health and Work Ability, Finnish Institute of Occupational Health, Helsinki, Finland; 5 Statistical Services Team, Finnish Institute of Occupational Health, Helsinki, Finland; University of Sydney, Australia

## Abstract

**Background:**

Sleep restriction, leading to deprivation of sleep, is common in modern 24-h societies and is associated with the development of health problems including cardiovascular diseases. Our objective was to investigate the immunological effects of prolonged sleep restriction and subsequent recovery sleep, by simulating a working week and following recovery weekend in a laboratory environment.

**Methods and Findings:**

After 2 baseline nights of 8 hours time in bed (TIB), 13 healthy young men had only 4 hours TIB per night for 5 nights, followed by 2 recovery nights with 8 hours TIB. 6 control subjects had 8 hours TIB per night throughout the experiment. Heart rate, blood pressure, salivary cortisol and serum C-reactive protein (CRP) were measured after the baseline (BL), sleep restriction (SR) and recovery (REC) period. Peripheral blood mononuclear cells (PBMC) were collected at these time points, counted and stimulated with PHA. Cell proliferation was analyzed by thymidine incorporation and cytokine production by ELISA and RT-PCR. CRP was increased after SR (145% of BL; *p*<0.05), and continued to increase after REC (231% of BL; *p*<0.05). Heart rate was increased after REC (108% of BL; *p*<0.05). The amount of circulating NK-cells decreased (65% of BL; *p*<0.005) and the amount of B-cells increased (121% of BL; *p*<0.005) after SR, but these cell numbers recovered almost completely during REC. Proliferation of stimulated PBMC increased after SR (233% of BL; *p*<0.05), accompanied by increased production of IL-1β (137% of BL; *p*<0.05), IL-6 (163% of BL; *p*<0.05) and IL-17 (138% of BL; *p*<0.05) at mRNA level. After REC, IL-17 was still increased at the protein level (119% of BL; *p*<0.05).

**Conclusions:**

5 nights of sleep restriction increased lymphocyte activation and the production of proinflammatory cytokines including IL-1β IL-6 and IL-17; they remained elevated after 2 nights of recovery sleep, accompanied by increased heart rate and serum CRP, 2 important risk factors for cardiovascular diseases. Therefore, long-term sleep restriction may lead to persistent changes in the immune system and the increased production of IL-17 together with CRP may increase the risk of developing cardiovascular diseases.

## Introduction

Sleep is generally considered to be a restorative process, having beneficial effects on immune functions. Partial loss of sleep is common among people who experience environmental or psychological stress such as travelling across time zones, having to do shift work, and in those individuals with psychiatric or physical disorders. Sleep restriction is becoming increasingly prevalent, especially among employed middle-aged populations [1], [2]. In modern 24-h societies, increased work demands are a major cause of chronic deficiency of sleep, leading to increased amounts of accidents, diseases, and even increased mortality [3]. It is important to understand the mechanisms by which sleep, immune responses and health are related if we are to find ways to manage patients with sleep disorders and people with chronically restricted sleep.

Sufficient sleep is vital for cardiovascular health and reduced sleep duration is specifically associated with increased cardiovascular morbidity [4]–[6]. Many cardiovascular risk factors, including heart rate, blood pressure and serum CRP concentrations, have been shown to increase during both short and prolonged periods of sleep restriction [6]. However, the underlying immunological mechanisms leading to the development of cardiovascular diseases remain to be elucidated.

In the present study, we simulated the accumulation of sleep loss during five working days followed by two days of weekend recovery sleep, and measured the changes in immunological parameters at these time points. We hypothesize that, in addition to the previously reported adverse effects on cognitive functioning and metabolism [7], [8], continuous sleep restriction disturbs human immunity which could result in an increased risk of developing cardiovascular diseases.

## Materials and Methods

### Participants and study design

Nineteen healthy men, aged 19–29 (mean [SD] age 23.1 [2.5] years), with a regular sleep-wake schedule and habitual sleep duration of 7–9 h participated in the study. Physical screening included blood tests (triglycerides, cholesterol, creatinine, haemoglobin, haematocrit, MCV, MCH, MCHC, leukocytes, erythrocytes, TSH, ASAT, ALAT) and polysomnography (Table S1). Two weeks prior to the experiment, participants completed sleep diaries, had an adaptation night in the sleep laboratory and wore actigraphs in order to verify adherence to a regular sleep-wake schedule. The pre-study mean (SD) sleep duration was 6.88 (0.58) h in the control group and 7.05 (0.80) h in the experimental group. The study design was approved by the ethics committee of Helsinki University Central Hospital, written informed consent was obtained from participants, and the experiment was conducted at the Brain and Work Research Centre of the Finnish Institute of Occupational Health.

The experimental group (n = 13) spent 8 h in bed for the first two nights (BL; from 11 PM to 7 AM), followed by 5 nights where they rested for only 4 h in bed (SR; from 3 AM to 7 AM) and, finally, again 3 nights of 8 h in bed (REC). The control group (n = 6) spent 8 h in bed (11 PM to 7 AM) throughout the entire experiment. Napping during daytime was not allowed and this was monitored by continuous EEG recordings and a continuously present investigator. Meals were standardized, provided at fixed times and finished by all participants throughout the experiment: breakfast at 8 AM (600 kcal), lunch at 12.30 PM (800 kcal), dinner at 6 PM (700 kcal); snacks at 3.30 PM (300 kcal) and 9.30 PM (200 kcal). In addition, participants in the experimental group ate a piece of fruit at 12.15 AM. Participants were not allowed to leave the building, but could stay in a living room where there was a television and a personal computer. Illumination in the sleeping room and in the test room ranged from 150 to 400 lux, and in the living room from 350 to 600 lux. The temperature ranged from 19 to 23°C.

### High sensitivity C-reactive protein (hs-CRP) and cortisol assays

Blood samples were taken from participants at 7.30 AM and analyzed by Medix Laboratories, Espoo, Finland for high sensitivity C-reactive protein (hs-CRP) using immunoturbidimetry. Morning peak values of cortisol were assessed using saliva samples that were collected 10 times a day and their cortisol levels were measured using a commercial kit assay (Salivary Cortisol, LIA, IBL, Hamburg, Germany). The measurement range was 0.43–110 nmol/L with assay repeatability values of 5% (within series) and 8% (between series).

### Heart rate and blood pressure measurements

ECG- based (WinAcq, AbsoluteAliens, Finland) heart rate together with continuous systolic and diastolic blood pressure (Portapres, Finapres Medical Systems, the Netherlands) was measured between 8 AM and 9 AM during a 10 minute period of rest.

### Peripheral blood mononuclear cells and flow cytometry

Peripheral blood mononuclear cells (PBMC) were isolated from heparinized venous blood by density gradient centrifugation as earlier described [9]. Cell distribution of PBMC was then assessed by flow cytometry (FACSCalibur, BD Biosciences, San Jose, CA, USA) using FACSComp software version 4.01 (BD Biosciences) [9]. Briefly, PBMC were incubated with (phycoerythrin-Cy5 (PE-Cy5)-conjugated anti-CD3 or with PE-conjugated anti-CD4, anti-CD56, anti-CD19, or with fluorescein isothiocyanate (FITC)-conjugated anti-CD8 or anti-CD14, and the corresponding isotype controls, which were all obtained from BD Biosciences. After incubation with monoclonal antibodies, cells were washed, and fixed in 1% paraformaldehyde (BD Biosciences). A total of 10000 events within the lymphocyte and monocyte gates was collected. The number of CD14+ cells (monocytes) within the monocyte gate and CD3+ (T cells), CD3+CD4+ (T helper cells), CD3+CD8+ (cytotoxic T cells), CD3-CD56+ (natural killer cells, NK cells) and CD19+ cells (B cells) within the lymphocyte population were analyzed by BD CELLQuestPro software (BD Biosciences).

### Proliferation assay of PBMC

Isolated PBMC were washed twice with phosphate buffered saline (PBS) and proliferation of PBMC was performed in complete RPMI 1640 containing 5% heat-inactivated human AB serum on 96-well U-bottomed plates (Costar Corning Incorporated, Corning, NY, USA) as earlier described [9]. Briefly, PBMC were cultured in triplicate (10^5^ cells/200 µL/well) alone or together with phytohaemagglutinin (PHA, 45 µg/mL; Murex Biotech Ltd, Dartford, UK). After 3 days of culture, tritiated methyl-thymidine ([methyl-^3^H]-TdR, Amersham Biosciences, Little Chalfont, UK) was added for 24 h. The results were calculated as stimulation indexes (uptake of isotype in stimulated culture/uptake of isotype in non-stimulated medium control culture) and expressed as percentages of individual baseline levels.

### Cytokine assays

Separated and washed PBMC were stimulated with PHA (45 µg/mL) in complete RPMI 1640 medium on 24-well plates (Costar) (3×10^6^ cells/1.5 mL/well). The total number of cells per stimulation was 6×10^6^. Cell pellets were collected after 6 h of incubation and used for RNA isolation. Cell culture supernatants were obtained after 24 h of incubation and stored at −70°C before measurement of cytokine protein levels.

Total RNA isolation, synthesis of cDNA and real-time quantitative PCR with an AbiPrism 7500 Fast Real-Time PCR System (Applied Biosystems, Foster City, CA, USA) were performed as described earlier [9]. Primers and probes for PCR were designed and purchased from Applied Biosystems. The results were calculated by the comparative CT method according to the instructions of Applied Biosystems using ribosomal 18 S as an endogenous control.

Cytokine protein analysis was made with the Luminex bead system (Bio-Plex 200 System, Bio-Rad Laboratories, Hercules, CA, USA) by labelled cytokine capture antibody pairs (Bio-Rad Laboratories).

### Statistical analysis

Data were expressed as percentages of each individual participant's baseline values unless otherwise specified. We have compared sleep restriction and recovery values to baseline values by applying paired t-tests for normally distributed differences and Wilcoxon signed ranks tests for differences that were not normally distributed. The normality of differences was checked using Kolmogorov-Smirnov goodness of fit test. A *P* value<.05 was considered to be statistically significant. All statistical analyses were carried out using SPSS version 15 (SPSS Inc., Chicago, USA).

## Results

### Cell distribution of PBMC

**Figure 1:**
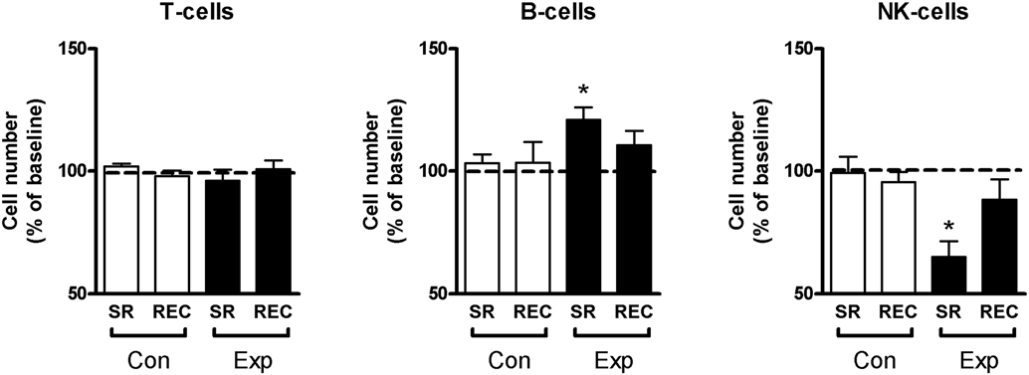
Cell distribution of PBMC. Number of T cells, B cells, and natural killer (NK) cells in PBMC, expressed as percentage of participant's individual baseline values. SR = sleep restriction, REC = recovery, Con = control group, Exp = sleep restriction group. Data are presented as mean values ±SEM. * *p*<0.05.

The cell distribution of peripheral blood describes the general immune status of these individuals. The total number of T cells (Figure 1) as well as the numbers of helper T-cells and cytotoxic T-cells did not change throughout the experiment either in the experimental or in the control group (Table S2). The number of NK-cells decreased significantly after the period of sleep restriction (to 65% of baseline levels, *p*<0.005), returning back to baseline levels after recovery (89% of baseline levels; Figure 1). The number of B cells increased significantly after the period of sleep restriction (to 121% of baseline levels, *p*<0.005) and returned to baseline levels again after recovery (111% of baseline levels; Figure 1). In the control group, the number of B-cells and NK-cells did not change throughout the experiment (Figure 1).

### CRP, Heart rate, blood pressure, and cortisol

**Figure 2:**
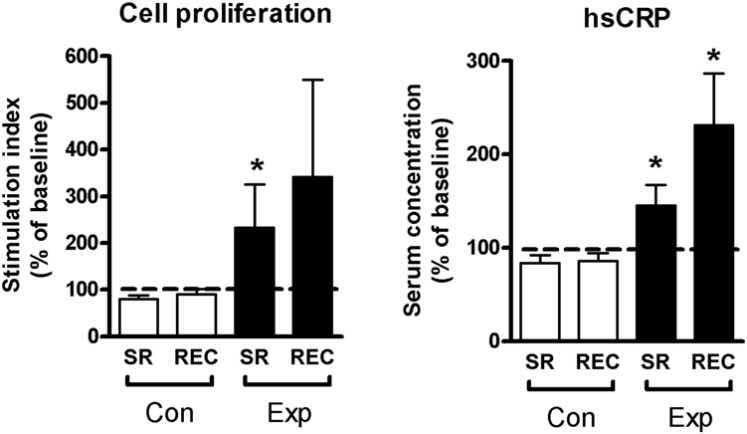
PBMC proliferation and hs-CRP. Proliferation of PBMC after stimulation with phytohaemagglutinin (PHA, 45 µg/mL) and high sensitivity C-reactive protein (hs-CRP) concentrations in plasma. SR = sleep restriction, REC = recovery, Con = control group, Exp = sleep restriction group. Data are expressed as percentages of participant's individual baseline values (mean±SEM). * *p*<0.05.

C-reactive protein (CRP) is an important risk factor for many diseases, including stroke and hypertension [10], [11]. Serum CRP increased significantly after sleep restriction (145% [*p*<0.05] of baseline levels) with the elevation being even more pronounced after recovery sleep (231% [*p*<0.05] of baseline levels; Figure 2). Heart rate increased throughout the experiment, reaching significance after recovery (108% [*p*<0.05] of baseline levels; Table S2). On the other hand, concentrations of stress hormone cortisol were not changed after sleep restriction (Table S2). Blood pressure values remained unaffected throughout the entire study (Table S2). In the control group, CRP, heart rate, cortisol, and blood pressure did not change during the experiment (Table S2).

### Proliferation of activated PBMC

Proliferation of PHA activated PBMC reflects the immunological capability of peripheral blood cells. Proliferation of PBMC in the experimental group was significantly increased after sleep restriction compared to baseline (to 233% [*p*<0.05] of baseline levels; Figure 2). After recovery sleep, there was still a tendency towards increased proliferation (341% [*p* = 0.53] of baseline levels). In contrast, cell proliferation in the control group decreased to 81% (*p*<0.05) and 90% (*p* = 0.35) of baseline levels, respectively (Figure 2).

### Cytokine production of PHA activated PBMC

**Figure 3:**
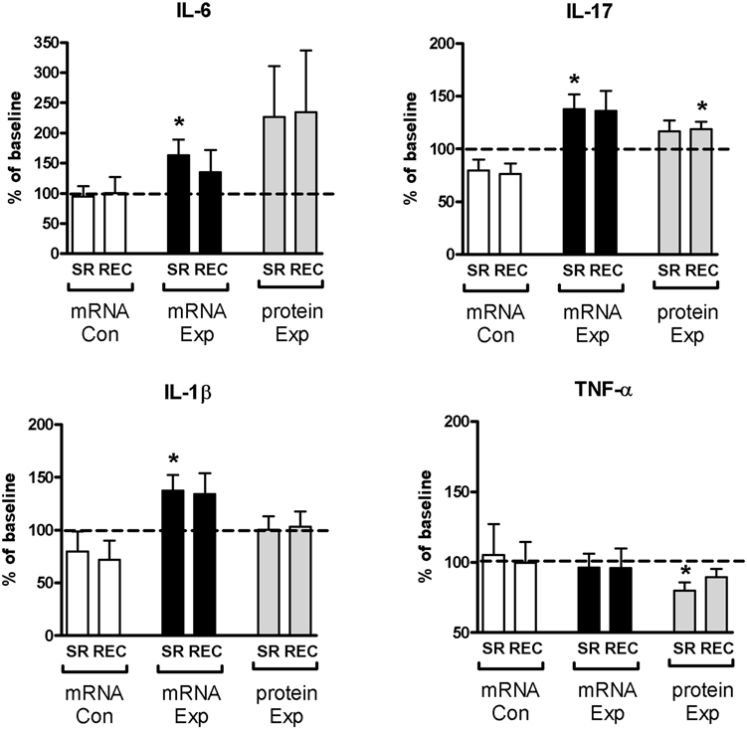
Cytokine mRNA and protein expression. Cytokine mRNA and protein expression of phytohaemagglutinin (PHA, 45 µg/mL) activated PBMC. SR = sleep restriction, REC = recovery, Con = control group, Exp = sleep restriction group. Data are expressed as percentages of participant's individual baseline values (mean±SEM). * *p*<0.05.

Cytokine profiles of activated PBMC reflect the dominating immune responses. The amounts of proinflammatory cytokines IL-1β and IL-6 increased significantly at the mRNA level (to 137% [*p*<0.05] and 163% [*p*<0.05] of baseline levels, respectively) whereas TNF-α decreased at the protein level after sleep restriction (to 80% [*p*<0.05] of baseline levels; Figure 3). After recovery sleep, concentrations of IL-6, IL-1β and TNF-α did not return to baseline levels completely. IL-17 showed significantly increased expression at mRNA level after sleep restriction (138% [*p*<0.05] of baseline levels) and at protein level after subsequent recovery sleep (119% [*p*<0.05] of baseline levels). The mRNA expression of all cytokines in the control group remained at the baseline level at all timepoints.

## Discussion

Chronic sleep deprivation is becoming increasingly common in modern, 24-h societies due to voluntary sleep restriction and increasing work demands [1], [2]. Sleep loss results in tiredness and impaired cognitive performance [8], but it also affects immune functions, leading to an increased number of infections [12], [13]. Moreover, it has been shown both epidemiologically and experimentally that reduced sleep duration is associated with an increased risk of developing diabetes [14], obesity [15], and cardiovascular diseases [4]–[6]. An accelerated heart rate is a sign of stress in the cardiovascular system [16]. In the present study, heart rate was significantly increased at the end of the experiment. This could be viewed as an alarm reaction in the circulatory system that might, at least partially, be a consequence of reduced parasympathetic tone and/or increased sympathetic tone in the autonomic nervous system (ANS). Serum cortisol and blood pressure remained unaffected throughout the current experiment, probably due to the fact that chronic changes in those parameters require more time to develop. Indeed, it has been shown that a more extended period of six nights of sleep restricted to 4 h per night results in elevated evening cortisol concentrations [17].

NK cells are phagocytes of innate immunity that quickly recognize, engulf and destroy intracellular pathogens whereas T cells and B cells orchestrate adaptive immunity through cellular and humoral responses. We observed a decrease in NK cell numbers as well as an increase in the number of B cells after five nights of sleep restriction. On the other hand, there were no changes in the number of T cells or their CD4+ and CD8+ subtypes. Sleep deprivation as well as stress factors have been shown to decrease the number and function of NK cells, often associated with increased susceptibility to infections [18], [19]. It should be noted, however, that the number of both NK cells and B-cells returned to baseline level during the two nights of recovery sleep in the present study. This suggests that the differences in the numbers of these cells in the circulation are probably due to reversible redistribution of these cells between lymphoid organs and periphery. In addition to cell numbers, the function of the immune cells plays a key role in successful immunity.

In our study, peripheral T cells showed highly elevated proliferation responses to PHA. This suggests that T cells in sleep deprived people, compared to people with normal sleep, are primed and after non-specific stimulation they respond more efficiently. A similar effect was observed in a recent study where stressed mice survived as well as or even better than non-stressed mice during a primary pneumococcus infection, but their survival was strongly reduced during secondary infection [20]. This was assumed to be due to a temporary immune-enhancing effect that later converted to a diminished adaptive immune response. In more extreme cases, chronic sleep loss in rats has resulted in severe inflammation in body tissues, culminating in lethal bacterial invasion of the bloodstream [21]. Therefore, over-activation of effector cells may enhance immunity and help the individual to survive through extraordinary conditions in the short term, but prolonging this situation leads to inflammation, and tissue injury.

CRP is widely used as a general marker for inflammation [10]. In the present study, serum hs-CRP concentrations were elevated immediately after sleep restriction and, since this peptide has an in vivo half life of 19 h [22], this elevation sustained after two days of recovery sleep. Similarly, a previous study has shown that both total (88 hours) as well as partial (4.2 hours during 10 consecutive nights) sleep restriction significantly increased serum concentrations of hs-CRP [6]. Elevated serum CRP is a risk factor for cardiovascular diseases and predicts future cardiovascular events and even mortality in apparently healthy people [10], [23]. CRP co-localizes with modified low density lipoprotein (LDL) in human atherosclerotic plaques, and it has been shown to increase platelet adhesion to endothelial cells. Therefore, in addition to acting as a biomarker, CRP plays a causal role in the development of atherosclerosis and thrombosis [11]. Animal studies also support the proinflammatory and pro-atherogenic role of CRP, because administration of human CRP or over-expression of CRP in apolipoprotein E -deficient mice promotes the development of spontaneous atherosclerosis [24]. Previously it has been reported that synthesis of CRP in the liver is controlled by proinflammatory cytokines, including IL-6, TNF-α and IL-1 [25]. In our study, the production of IL-6 and IL-1β was clearly increased by PHA activation of PBMC after sleep restriction and remained elevated after recovery sleep, whereas the production of TNF-α was slightly reduced, but recovered after two days of recovery sleep. Cytokines IL-1 and IL-6 play a crucial role in immune defenses and their secretion also regulates sleep-wake rhythms and sleep patterns, respectively [26]. In line with the results of our study, it was recently shown that IL-6 increased during prolonged sleep restriction when subjects slept only 4 hours per night for 12 days [27]. Independently of high cholesterol or myocardial damage markers, IL-6 predicts future adverse cardiovascular events and reflects increased inflammatory activity in plaques and is therefore a strong marker of increased risk for mortality in coronary artery diseases [28], [29]. IL-1β is a proinflammatory cytokine; the processing of its inactive form (pro-IL-1β) to the active form is triggered by microbial products and metabolic stress, leading to increased lymphocyte activation and destruction of local tissues. Thus, secretion of biologically active IL-1β protein may be induced when sleep deprived people are infected (i.e. enhanced susceptibility to viral and bacterial infections), a phenomenon which is frequently associated with chronic sleep deprivation.

**Figure 4:**
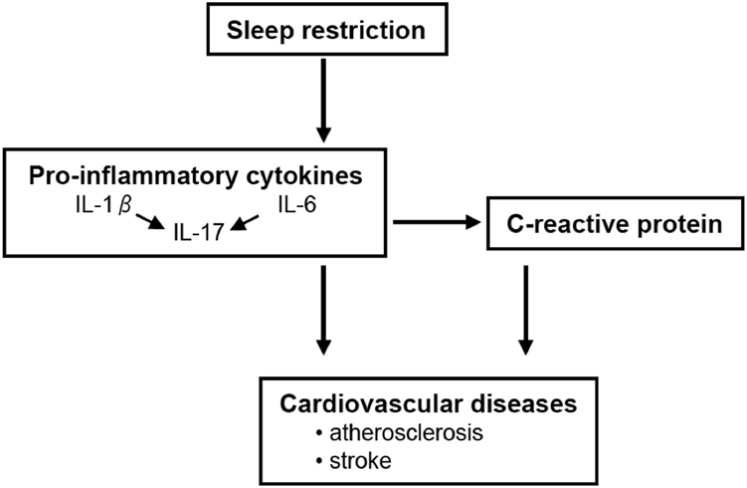
From sleep restriction to cardiovascular diseases. Schematic flow chart showing the proposed mechanism explaining how sleep restriction may ultimately evoke increased cardiovascular morbidity.

In the present study, five days of sleep restriction were associated with increased IL-17 production both at the mRNA and the protein levels from PHA activated PBMC, and the amount of IL-17 remained elevated after the recovery period. IL-17 is a relatively newly-discovered member of the proinflammatory cytokines. It plays a key role in sustaining tissue damage in several tissues such as brain, joints, heart, lung and intestine, sometimes promoting the development of autoimmune diseases [30]. Inflammation is an important component in all stages of atherosclerosis and interestingly, IL-17 has recently been reported to stimulate expression of CRP in hepatocytes and in coronary artery smooth muscle cells [31]. On the basis of these results, we propose a hypothesis for the sleep restriction-induced development of cardiovascular diseases (Figure 4). Prolonged sleep restriction results in activation of the synthesis of proinflammatory cytokines IL-1β and IL-6 which in turn increases the expression of IL-17. These cytokines play an important role in the induction of CRP which may facilitate, directly or indirectly, the formation of atherosclerotic plaques leading to an increased risk for cardiovascular diseases. This detrimental pathway may be even further activated by simultaneous exposure to a microbial infection. However, understanding of the mechanisms of IL-17 in bridging innate and adaptive immunity is still in its infancy, and therefore the specific role of IL-17 in the development of cardiovascular diseases needs to be studied in more detail.

In conclusion, we identified how prolonged sleep restriction can change immune cell functions, and may lead to an increased risk to develop cardiovascular diseases. Several immunological changes that occurred after five days of sleep restriction did recover after two nights of normal sleep, but the elevated level of serum hs-CRP that was accompanied by increased production of proinflammatory cytokines, especially IL-17, did not return to normal. In summary, these results indicate that immunological changes that take place after multiple nights of short sleep cannot be restored completely by sleeping normally for a few nights, and long-term sleep restriction may lead to an increased risk of developing cardiovascular diseases.

## Supporting Information

Table S1 Pre-study screening results
(0.08 MB DOC)

Table S2 Overview of the Results of All Variables with Paired T-tests: Comparison to Baseline
(0.08 MB DOC)
