# Supplementary material for: Sleep Restriction Increases the Risk of Developing Cardiovascular Diseases by Augmenting Proinflammatory Responses through IL-17 and CRP
Source: PLoS One. 2009 Feb 25;4(2):e4589. doi: 10.1371/journal.pone.0004589 (PMC2643002; doi:10.1371/journal.pone.0004589)
Supplement: Table S1 — Pre-study screening results (0.08 MB DOC) [file pone.0004589.s001.doc]

**Table S**1

| **Parameter** | **Mean (±SEM)** |
| --- | --- |
| B-Leukocytes (x 10^9^/l) | 6.0 (±0.4) |
| B-Erythrocytes (x 10^12^/l) | 4.9 (±0.1) |
| B-Haemoglobin (g/l) | 149.9 (±1.5) |
| B-Haematocrit (%) | 45.2 (±0.6) |
| E-MCV (fl) | 92.4 (±0.8) |
| E-MCH (pg/cell) | 30.6 (±0.3) |
| E-MCHC (g/l) | 331.8 (±1.6) |
| S-ASAT (U/l) | 26.0 (±1.7) |
| S-ALAT (U/l) | 28.9 (±3.9) |
| fS-Cholesterol (mmol/l) | 4.3 (±0.1) |
| fS-Triglycerides (mmol/l) | 0.9 (±0.1) |
| S-TSH (mU/l) | 2.0 (±0.1) |
| U-Creatinine (mmol/l) | 19.9 (±2.2) |
