# Supplementary material for: Sleep Restriction Increases the Risk of Developing Cardiovascular Diseases by Augmenting Proinflammatory Responses through IL-17 and CRP
Source: PLoS One. 2009 Feb 25;4(2):e4589. doi: 10.1371/journal.pone.0004589 (PMC2643002; doi:10.1371/journal.pone.0004589)
Supplement: Table S2 — Overview of the Results of All Variables with Paired T-tests: Comparison to Baseline (0.08 MB DOC) [file pone.0004589.s002.doc]

## Table S2

| **Group** | **Variable** | **Day** | | | |
| --- | --- | --- | --- | --- | --- |
|  |  | **SR (BL = 100)** | | **REC (BL = 100)** | |
|  |  | **Mean (SD)** | **P** | **Mean (SD)** | **P** |
| Control | Cortisol | 96.2 (22.8) | 0.65 | 98.0 (17.9) | 0.76 |
|  | Heart rate | 102.2 (9.5) | 0.53 | 103.5 (8.2) | 0.27 |
|  | Systolic blood pressure | 94.4 (23.0) | 0.51 | 97.9 (14.1) | 0.68 |
|  | Diastolic blood pressure | 103.2 (14.2) | 0.55 | 98.7 (12.5) | 0.78 |
|  | T-cells | 102.0 (2.7) | 0.13 | 98.1 (5.2) | 0.40 |
|  | Helper T-cells | 97.1 (4,3) | 0.16 | 94.1 (7.7) | 0.12 |
|  | Cytotoxic T-cells | 101.4 (18.9) | 0.86 | 95.7 (22.3) | 0.65 |
|  | Monocytes | 86.5 (26.8) | 0.27 | 78.1 (18.0) | < 0.05 |
|  | B-Cells | 103.2 (8.9) | 0.41 | 103.3 (21.1) | 0.71 |
|  | NK-cells | 99.3 (15.6) | 0.92 | 95.5 (10.3) | 0.34 |
|  | hsCRP | 83.9 (20.2) | 0.11^*^ | 86.2 (20.2) | 0.11^*^ |
|  | Proliferation | 80.7 (19.5) | < 0.05^*^ | 90.3 (26.9) | 0.35^*^ |
|  | IL-6 mRNA | 95.0 (38.3) | 0.79 | 100.1 (61.0) | 0.99 |
|  | IL-1β mRNA | 79.7 (42.1) | 0.34 | 71.9 (40.3) | 0.19 |
|  | IL-17 mRNA | 79.8 (20.7) | 0.15 | 76.4 (20.2) | 0.10 |
|  | TNF-α mRNA | 105.1 (49.4) | 0.83 | 99.7 (33.2) | 0.98 |
| Experimental | Cortisol | 105.8 (25.7) | 0.43 | 103.1 (39.9) | 0.78 |
|  | Heart rate | 103.6 (6.6) | 0.06 | 108.2 (8.7) | < 0.05 |
|  | Systolic blood pressure | 97.0 (12.5) | 0.36 | 98.2 (11.6) | 0.58 |
|  | Diastolic blood pressure | 102.4 (15.0) | 0.55 | 99.2 (16.4) | 0.86 |
|  | T-cells | 96.1 (15.9) | 0.40 | 100.8 (12.5) | 0.83 |
|  | Helper T-cells | 99.7 (14.6) | 0.95 | 102.1 (16.6) | 0.68 |
|  | Cytotoxic T-cells | 94.7 (24.2) | 0.45 | 99.7 (15.4) | 0.94 |
|  | Monocytes | 103.9 (36.6) | 0.71 | 122.2 (38.2) | 0.07 |
|  | B-Cells | 121.0 (18.2) | < 0.005 | 110.7 (20.0) | 0.09 |
|  | NK-cells | 65.2 (23.2) | < 0.001 | 88.5 (28.1) | 0.19 |
|  | hsCRP | 145.2 (85.7) | < 0.05^*^ | 231.1 (208.4) | < 0.05^*^ |
|  | Proliferation | 233.0 (336.2) | < 0.05^*^ | 341.2 (723.7) | 0.53^*^ |
|  | IL-6 mRNA | 162.9 (94.1) | < 0.05 | 135.5 (125.6) | 0.35 |
|  | IL-6 protein | 226.7 (222.4) | 0.13^*^ | 234.9 (269.7) | 0.31^*^ |
|  | IL-1β mRNA | 137.4 (54.0) | < 0.05 | 134.2 (69.0) | 0.11 |
|  | IL-1β protein | 100.6 (35.5) | 0.96 | 103.0 (41.8) | 0.84 |
|  | IL-17 mRNA | 137.9 (50.4) | < 0.05 | 135.8 (67.0) | 0.09 |
|  | IL-17 protein | 116.7 (29.3) | 0.15 | 118.7 (20.5) | < 0.05 |
|  | TNF-α mRNA | 96.3 (35.7) | 0.71 | 96.0 (48.4) | 0.78 |
|  | TNF-α protein | 80.0 (16.1) | < 0.05 | 89.3 (16.7) | 0.11 |
| ^*^ Wilcoxon signed ranks test has been applied, since variable was not normally distributed | | | | | |
